# Does HIV infection increase male sexual behavior?

**DOI:** 10.1093/emph/eoaa030

**Published:** 2020-09-16

**Authors:** Philip T Starks, Maxfield M G Kelsey, David Rosania, Wayne M Getz

**Affiliations:** e1 Department of Biology, Tufts University, Medford, MA 02155, USA; e2 PO Box 93, Rye Beach, NH 03871, USA; e3 University of California at Berkeley, Berkeley, CA 94720-3112, USA; e4 School of Mathematical Sciences, University of KwaZulu-Natal, Private Bag X54001, Durban 4000, South Africa

**Keywords:** parasite, host, manipulation, AIDS, acute, early stage

## Abstract

After 40 years of intense study on HIV/AIDS, scientists have identified, among other things, at risk populations, stages of disease progression and treatment strategies. What has received less attention is the possibility that infection might elicit an increase in sexual behavior in humans. In 2000, Starks and colleagues speculated that HIV infection could alter host behavior in a manner that facilitated the spread of the virus. Retrospective and self-report data from five studies now support this hypothesis. Individuals with acute—versus nonacute—stage infections report more sexual partners and more frequent risky sex. Additionally, male sexual behavior increases nonlinearly with HIV viral load, and data suggest a potential threshold viral level above which individuals are more likely to engage in risky sexual behavior. Taken together, these data suggest that HIV infection influences male sexual behavior in a manner beneficial to the virus. Here, we present these findings, highlight their limitations and discuss alternative perspectives. We argue for increased testing of this hypothesis and advocate for increased public health measures to mitigate the putative impact on male sexual behavior.

**Lay Summary** In 2000, Starks and colleagues speculated that HIV infection could alter host behavior in a manner that facilitated the spread of the virus. Retrospective and self-report data from five studies now support this hypothesis. We argue for increased testing of this hypothesis and advocate for increased public health measures to mitigate the putative impact on male sexual behavior.

## STAGES OF HIV INFECTION

The progression of HIV infection is divided into distinct stages marked by differences in serology, viral load, and CD4+ cell counts. Acute infections are active during the period ∼2–5 weeks after transmission, with the production of HIV-specific antibodies commencing around 3–4 weeks after transmission [1]. Like the acute stage, the early stage begins at infection, but it continues until viral load is reigned into its set point between 2 and 3 months after transmission [1]. The chronic stage has different presentations depending on whether antiretroviral therapy is ongoing, but it lies between the early and late stages and exhibits intermediate viral levels. This stage lasts anywhere from 2 to 20 years in untreated individuals and can be lifelong in treated individuals. The late stage, or AIDS, is the final phase and is diagnosed if CD4+ cell count is <200 cells/mm^3^ or if certain opportunistic infections are present. As in the acute stage, viral load is often high during AIDS [2–4]. Our hypothesis deals specifically with the acute and early stages when viral load is especially high, but when individuals may be unaware of HIV status.

## OUR HYPOTHESIS AND SUPPORTING DATA

A 2016 review [5] notes that there exists a paucity of research into human behavioral manipulation by sexually transmitted infections. We hope that this article can encourage a course correction in this situation. We hypothesize that during the acute and early stages of HIV infection, the virus manipulates host behavior such that it increases male sexual behavior, resulting in more frequent and riskier sex with more partners [6]. While this topic may be perceived as controversial, and the topic of masculine behavior and HIV status complicated, the hypothesis rests upon established science: disease phenomena subject to selection include alteration of host behavior by infectious agents [7–9]. Human behavioral manipulation by a virus is not without precedent. Rabies induces hypersalivation and stereotyped aggression in its mammalian hosts, thus increasing the likelihood it will be spread through biting or scratching. As is observed in cases of furious rabies, humans display hyperactivity and hydrophobia clearly demonstrating that we experience behavioral manipulation by pathogens [10, 11]. We are in no way implying that HIV+ individuals exhibit glaring changes in behavior, however: Our hypothesis suggests a much subtler effect, mediated by unconscious processes.

Directly testing our hypothesis is challenging. Assessment of altered sexual behavior caused by HIV infection requires observation before and after infection. A randomized controlled trial would require an at-risk group to be monitored for an extended period. Given the potentially confounding impacts of both knowledge of infection and treatment with antiretrovirals, individuals would need to remain unaware of their infection and remain untreated. For obvious ethical reasons, such an experimental design cannot be implemented. It is possible, however, to examine sexual behavior of HIV+ individuals as related to stage of infection or viral loads. In particular, stage of infection studies can offer similar experimental benefits to the aforementioned morally problematic design. Here, we use retrospective and self-report data from five studies to examine our hypothesis.

Joseph Davey *et al*. [12] found that at their time of HIV+ diagnosis, homosexual men with acute infections reported an average of 4.2 sexual partners during the previous month (−1 M) and an average of 2.85 sexual partners per month in the two antecedent months (−3 M and −2 M). Since the acute stage lasts ∼24 days following infection, at most a fraction of the previous month was spent infected for almost all acute stage individuals [4]. Had sexual behavior been unchanged by infection, the −3 M −2 M average would be equal to the −1 M average. This is not the case, and the magnitude of the inequality suggests that the average number of sexual partners substantially increases following HIV infection. The median number of sexual partners reported during these periods tells a similar story (Table 1).

**Table 1.** Behavioral risk factors for HIV acquisition among men who have sex with men, for whom HIV infection was diagnosed at the Los Angeles Lesbian, Gay, Bisexual, and Transgender center during 2011–2015, by diagnosis. This caption and table were originally published by Joseph Davey *et al*. [12, table 2].

Risk Factor	Acute Infection (*n* = 145)	Nonacute Newly Diagnosed Infection (*n* = 764)
Sex partners, no., by time		
Past 30 d		
Mean ± SD	4.2 ± 6.2^a^	2.4 ± 5.3
Median	2.0	1.0
Past 3 mo		
Mean ± SD	9.9 ± 17.6^a^	5.3 ± 10.1
Median	4.0	2.0
Prevalence of condomless anal intercourse in past 3 mo, subjects, no. (%), by intercourse type		
Receptive	71 (65.1)^b^	396 (55.8)
Insertive	60 (55.1)	355 (50.0)
Abbreviation: SD, standard deviation		

a *P* < 0.001, by the Wilcoxon rank sum test, compared with subjects with nonacute newly diagnosed infection.

b Crude odds ratio, 1.85 (95% confidence interval, .99–3.46); *P* = .05.

Joseph Davey *et al*. [12] also found that men with acute infections reported on average twice the number of sexual partners in the previous month compared to men with nonacute infections (Table 1). The effect was strong enough to persist even when averaged over the previous 3 months. Condomless receptive and insertive behavior was also more frequent when averaged over the previous 3 months for men with acute infections: 65.1% versus 55.8% for condomless receptive behavior and 55.1% versus 50.0% for condomless insertive behavior. In agreement with Joseph Davey *et al*. [12], Braun *et al*. [13] report that diagnosis with an acute (*n =* 169) compared with nonacute (*n =* 5015) HIV infection in homosexual men was associated with 5-fold higher odds for future risky sexual behavior, which they defined as condomless sex with an occasional partner (adjusted odds ratio [OR], 5.58).

**Figure 1.**
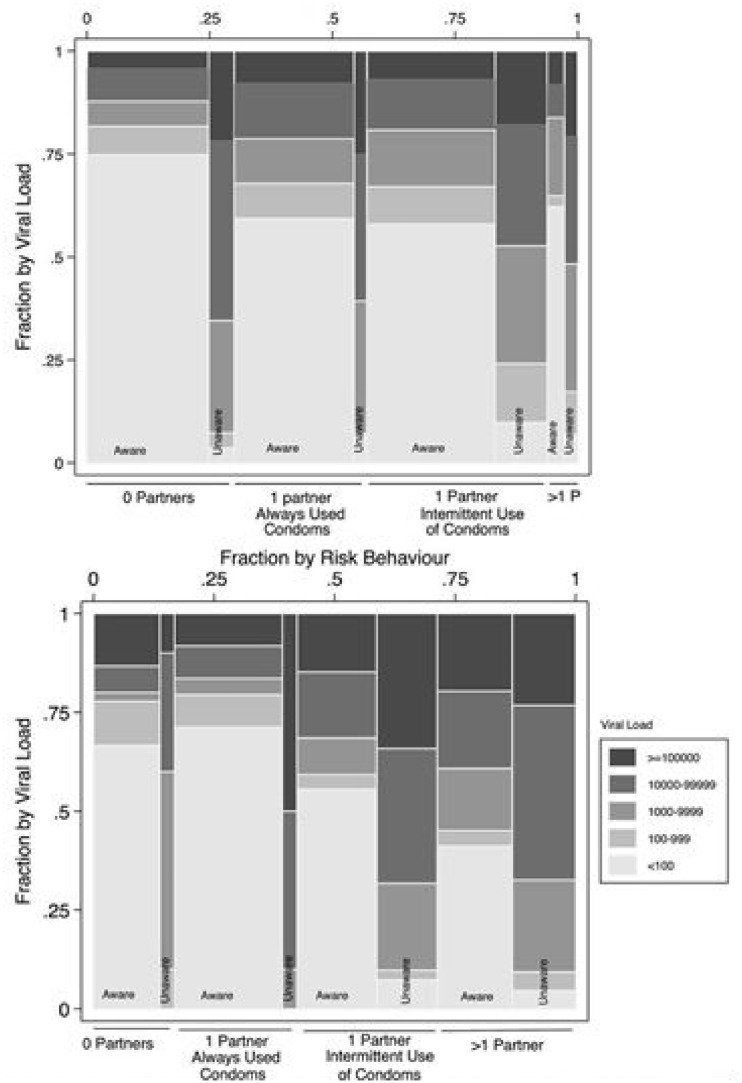
Blood viral load according to the sexual behavior and HIV status awareness among 338 men (top) and 1085 women (bottom) participating in the South African National HIV Prevalence, Incidence and Behaviour Survey, 2012 [14]. The width of the columns represents the proportion of individuals in the group. This figure was originally published by Huerga *et al*. [14, figure 2, CC BY 4.0].

Huerga *et al*. [14] found that in men, who were aware of their HIV+ status, increases in blood viral load (high viral load being indicative of an acute/early infection or of AIDS) were correlated with engaging in riskier sex practices, which they defined as inconsistent condom use during vaginal or anal intercourse, and in having greater number of sexual partners (Fig. 1). Dukers and colleagues (2001) found a relationship between the rate of unprotected sex and viral load, this time in serum. Their data show that the rate of unprotected sex with casual partners dips slightly from just over to just under 30% as the HIV-1 RNA load rises from 10^2^ to 10^4^ copies/ml and then rises parabolically to around 80% at between 10^6^ and 10^7^ copies/ml (See Fig. 3 in [15]). When subdivided, there is strong trend (OR=1.9; p=0.07) toward increased unprotected sex with casual partners with a recent increase in serum viral load above 10^5^ copies per ml. Continued elevated viral load was not significantly related to riskier sexual practices with casual partners, but the rate of unprotected sex was still elevated (OR=1.5). Consistent with the findings presented by Joseph Davey *et al*. [12], this suggests that the virus preferentially increases sexual behavior when the host is most infectious, which is in the early stages of infection [16]. This accords with the finding that about half of all HIV transmission occurs from an individual in the acute phase [4]. Furthermore, a recent phylogenetic study of HIV-1 transmission pairs shows that more founder variants of HIV-1 are transmitted during acute infection than during chronic infection [17]. This suggests that hosts are especially infectious during this early period of infection.

**Figure 2.**
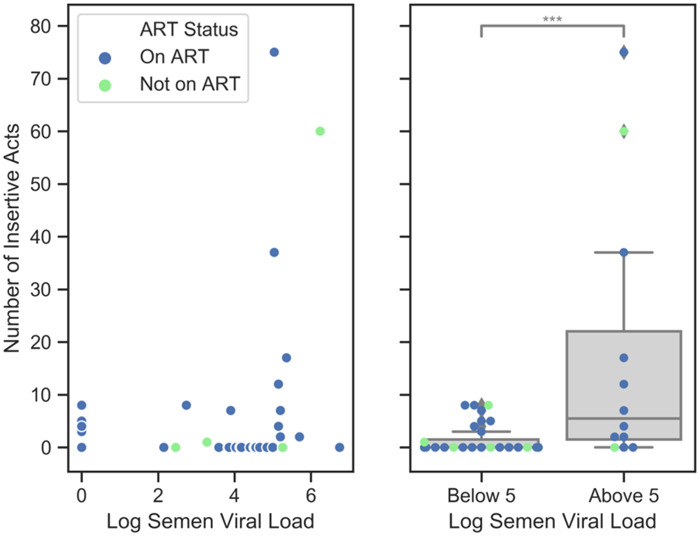
Number of insertive sexual acts (anal, vaginal and oral) engaged in during the preceding 3 months according to the log of semen viral load for 44 HIV-infected men (*U* = 87.5, *P* = 0.001). This community-based study was conducted in the year 2000 (data reanalyzed from Kalichman *et al*. [18, table 2]).

Kalichman *et al*. [18] found that insertive sexual behavior, which is most infectious, significantly increased with viral load in semen [19]. It appears that 10^5^ viral copies/ml is a threshold level above which the number of insertive acts rapidly increases. Stratifying semen viral loads by less than or greater than 10^5^ copies/ml shows a significantly greater frequency of insertive sexual acts in the group with higher viral loads (Fig. 2). Their analysis also revealed that having a greater viral load in semen relative to plasma was significantly associated with reporting a greater total number of unprotected insertive sexual intercourse acts.

Together these data suggest that HIV infection increases male sexual behavior during the acute and early stages, when an individual is especially infectious. At this time, we remain agnostic as to the proximate mechanism influencing male sexual behavior. HIV can potentially modulate all the endocrine systems and it infects cells in the brain [20, 21]. We note, however, that increased levels of testosterone, an endocrine mediator of sexual behavior and immunocompetence, have been observed in HIV+ men without AIDS [6, 22–24].

## DATA LIMITATIONS

Certain findings by Joseph Davey *et al*. [12] require further interpretation. A comparison between the number of sexual partners reported by acute stage individuals when they were assumed to be uninfected (−3 M, −2 M) and infected nonacute stage individuals suggests that nonacute individuals might have a depressed sexual frequency relative to uninfected individuals (Table 1). Alternatively, it could be the case that the −3 M, −2 M period is marked by an elevated sexual frequency in the acute cohort. These alternatives are of course not mutually exclusive. To the degree the former explanation is correct, the comparison between acute versus nonacute number of sex partners is confounded.

The number of sexual partners reported by Joseph Davey *et al*. [12] in each group is, as one might expect, highly variable; the coefficients of variation for the acute stage average number of sexual partners during the previous month and the previous 3 months are 1.47 and 1.77, respectively (Table 1). The 95% confidence intervals for the previous month average and for the previous 3-month, per-month average have substantial overlap.

The behavioral time frame assessed by Braun *et al*. [13] is lengthy (1.5 years) relative to the suspected window of behavioral manipulation by HIV. The poor temporal resolution means we cannot be certain that the increase in risky behavior occurred during, and not following, the acute/early stage. The temporal resolution of the Joseph Davey *et al*. [12] findings is better (previous month and previous 3-month averages are reported), though not so focused as to be considered optimal for our purposes. Ideally, there would be a weekly statistic, though such temporally resolved figures might diminish the accuracy of self-reports.

Individuals with AIDS exhibit hypogonadism and considerable sexual dysfunction [8, 25–27]. Since the studies we analyzed that measured viral load [14, 15, 18] do not distinguish between individuals in the acute or final stage, the strength of the signal we are examining, namely an increase in sexual behavior accompanying high viral load during the acute and early stages, is almost certainly attenuated. This may explain why the association between risky sex and blood viral loads has been inconsistently reported [28].

Data presented by Huerga *et al*. [14] in Fig. 1 were unevenly distributed when grouped by sexual risk behaviors; certain groups had sample sizes that prevented rigorous statistical analysis. Though not statistically prohibitive, the sample size in Kalichman *et al*. [18] was small (*n =* 44 HIV+ men). In sum, although supporting our hypothesis, all five referenced articles suffer from some limitations.

## ALTERNATIVE PERSPECTIVES

It can be argued that Joseph Davey *et al*.’s data [12] listed in Table 1, showing increased sexual activity in men diagnosed with acute versus nonacute HIV infections, are confounded. First, individuals are more likely to be infected with HIV when engaging in more frequent risky sex and it would be expected that the behavior would continue beyond the infection event. This could produce the observed pattern as an artefact. If true, however, this would predict that the period preceding infection would demonstrate the same pattern, which is not the case. Nevertheless, the temporal resolution afforded by these data is weak for our purposes and we contend that a 3-month window might well contain relevant changes in context.

Dukers *et al*. [15], Huerga *et al*. [14] and Kalichman *et al*. [18] did not observe sexual behavior before and after infection. This leaves open the possibility that extraneous variables can account for both the observed higher viral load and the higher sexual behavior. Sickness behavior is a constellation of behavioral changes following infection which can include reduced libido [29, 30]. Perhaps a more limited immune response in some individuals could cause higher viral loads and reduced sickness behavior leading to greater observed sexuality. Alternatively, perhaps the subset of HIV+ individuals who choose to not undergo antiretroviral treatment have a higher baseline sexuality.

These are but two of several potential explanations for the observations raised by these studies. However, the suggested alternate explanations seem less plausible than our hypothesis both *a priori* and when assessed in light of the findings on sexual partner diversity and risk taking in uninfected, acute stage and nonacute stage individuals reported by Joseph Davey *et al*. [12] and Braun *et al*. [13].

## SUGGESTIONS

We believe that future research on HIV and its potential impact on sexual behavior should examine semen viral loads in addition to blood viral loads since these only weakly correlate [18]. Blood viral loads give a systemic view of infection while semen viral loads are more predictive of sexual infectiousness. It would seem most effective for a virus to increase the frequency of risky sexual behavior when its host was most infectious, and accordingly, we speculate that semen viral load may better predict behavioral manipulation.

Additionally, there are at least four broad groups of HIV-1 (M, N, O and P) which are further distinguished into subtypes that can have different biological effects [31]. We suggest that future research pay attention to the human and viral genetic backgrounds under investigation: it may be the case that manipulation only occurs in one such background or intersection of backgrounds.

Finally, HIV-1 is a relatively new human pathogen that originates from African primates; it first infected humans about 100 years ago [32, 33]. Accordingly, the vast majority of this viral lineage’s evolution occurred in simian hosts. If HIV is behaviorally manipulating humans, then it is possible that this behavioral manipulation evolved in its previous hosts. It would be informative to determine whether viral sexual manipulation is correlated with the length of the evolutionary relationship between the virus and its host or with any particular species characteristic (e.g. mating system). Directly testing our hypothesis in primates that develop immunodeficiencies is ethically fraught. Experimental work might be considered in primates whose simian immunodeficiency virus infection does not tend to produce AIDS like pathology, such as sooty mangabeys or African green monkeys [34]. We would also urge any unpublished observational data bearing on the matter be released.

## CONCLUSION

The fact that pathogens can influence the behavior of hosts is well understood. It stands to reason that sexual behavior is the most likely trait for a sexually transmitted virus to manipulate, and data show that risky sexual behavior increases following infection during the acute stage and with increasing viral loads. The data upon which we have based our analysis are limited. Consequently, we hope to spur further research that would more definitively support or reject our hypothesis. Seeing as knowledge of infection seems able to curb risky sexual behavior, our hypothesis argues strongly in favor of frequent screening in order to catch infections early on when they are most infectious and manipulative [35–38]. In addition, individuals at significant risk for HIV transmission might be encouraged to self-monitor for increased libido, sexual frequency and unprotected sex as possible proxies for infection, and therefore to seek testing. Awareness of the mechanisms underpinning alleged behavioral alterations induced by infection could better allow us to devise strategies to combat this effect and hinder disease transmission.
